# Fire‐Safe Hierarchical Solid Polymer Electrolytes Enabled by Dual‐Functional Microcapsules for Lithium Metal Batteries

**DOI:** 10.1002/advs.78034

**Published:** 2026-10-04

**Authors:** Ziqi Huang, Jiuqing Gui, Qiaoying Cao, Yong Xiao, Hang Hu, Yeru Liang

**Affiliations:** ^1^ College of Materials and Chemical Engineering, Key Laboratory for Biobased Materials and Energy of Ministry of Education South China Agricultural University Guangzhou People's Republic of China

**Keywords:** active fire protection, ammonium hydrogen phytate, dual‐functional fire‐safe microcapsules, hierarchical solid polymer electrolytes, lithium metal batteries

## Abstract

Lithium metal batteries offer high energy density but suffer from severe safety risks associated with dendrite growth and flammable electrolytes, which can lead to thermal runaway and combustion. Although solid‐state electrolytes improve thermal stability, they primarily rely on intrinsic nonflammability and remain ineffective in suppressing combustion once thermal runaway is triggered. Here, we report a dual‐functional fire‐safe microcapsule‐enabled strategy to construct hierarchical solid polymer electrolytes (HSPEs) with integrated fire‐suppression capability. Fire‐extinguishing agents, perfluoro(2‐methyl‐3‐pentanone), are encapsulated within ammonium hydrogen phytate‐modified urea‐formaldehyde‐based resin shells and incorporated into an ethylene‐vinyl acetate matrix. Upon thermal stimulation, the microcapsules undergo rapid structural rupture, releasing perfluoro(2‐methyl‐3‐pentanone) to suppress combustion, while the ammonium hydrogen phytate‐derived shell forms a protective char layer that retards heat and mass transfer. This dual‐functional design enables thermally triggered fire suppression within the electrolyte framework. Importantly, the HSPEs maintain desirable electrochemical properties, including a room‐temperature ionic conductivity of 0.64 mS cm^−1^ and an electrochemical stability window of up to 4.6 V. Furthermore, pouch cells based on HSPEs effectively resist ignition under thermal abuse conditions. This work provides a viable approach to integrating fire‐suppression functionality into solid‐state electrolytes, offering new insights into the design of safer lithium metal batteries.

## Graphical abstract

Dual‐functional fire‐safe microcapsules are integrated into hierarchical solid polymer electrolytes to provide sequential protection against thermal abuse. The ammonium hydrogen phytate‐modified shell forms a protective char barrier, while the thermally triggered release of perfluoro(2‐methyl‐3‐pentanone) actively suppresses combustion, enabling safer solid‐state lithium metal batteries without compromising electrochemical performance.

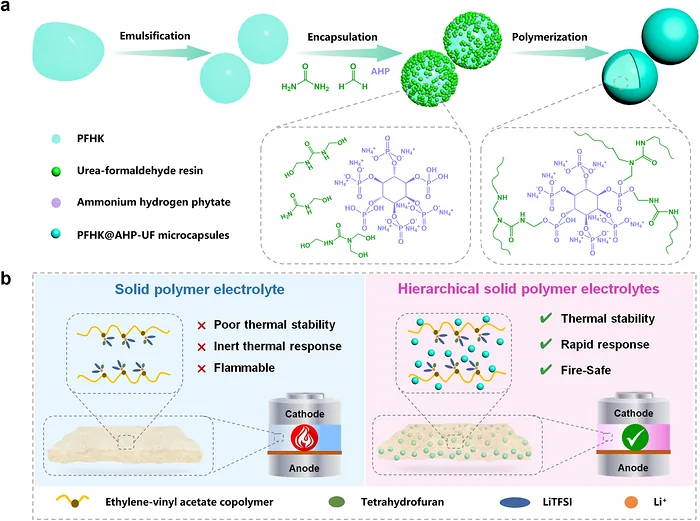

## 1 Introduction

Lithium (Li) metal has long been regarded as one of the most promising anode materials for next‐generation rechargeable batteries owing to its ultralow electrochemical potential (‐3.040 V vs. standard hydrogen electrode) [1, 2, 3] and exceptionally high theoretical specific capacity (3860 mAh g^−1^) [4]. These intrinsic advantages enable lithium metal batteries (LMBs) to deliver substantially higher energy density [5, 6, 7], making them attractive for advanced energy storage applications ranging from electric vehicles to grid‐scale storage. However, the practical realization of high‐energy LMBs is persistently hindered by critical safety concerns [8, 9]. During repeated Li plating/stripping, nonuniform deposition induces dendritic growth that may penetrate separators and trigger internal short circuits [10, 11]. Meanwhile, the presence of flammable liquid electrolytes further exacerbates thermal hazards, as interfacial instability or short‐circuiting can rapidly lead to heat accumulation and thermal runaway [12, 13].

Replacing flammable liquid electrolytes with solid‐state electrolytes represents a promising pathway toward intrinsically safer LMBs [14, 15, 16, 17]. Benefiting from their superior thermal stability [18, 19, 20, 21] and mechanical robustness [22, 23, 24], solid‐state electrolytes can suppress dendrite propagation and mitigate leakage or volatilization risks. Nevertheless, safety under abusive conditions remains far from guaranteed. Once thermal runaway is initiated, exothermic reactions within the cell can still accelerate temperature rise and induce catastrophic failure. Notably, most solid‐state systems rely primarily on intrinsic nonflammability to resist ignition [25, 26, 27, 28], yet lack the capability to effectively suppress combustion once it occurs.

To address these challenges, substantial efforts have been devoted to engineering electrolyte systems in solid‐state LMBs. One widely explored strategy is the incorporation of flame‐retardant additives into solid‐state electrolytes, particularly polymer‐based electrolytes [29, 30, 31]. Typical flame retardants include phosphorus‐containing compounds [32, 33], fluorinated solvents [34, 35, 36], and fluorinated phosphazenes [37]. These additives suppress combustion primarily via free‐radical quenching and gas‐phase inhibition, thereby interrupting chain reactions during burning [38, 39]. However, their effectiveness generally relies on already‐initiated combustion processes, rendering their flame‐retardant function largely passive. Beyond conventional flame‐retardant additives, thermally responsive materials have been introduced to regulate battery behavior under abnormal conditions, including thermoresponsive polymers [40], phase‐change materials [41, 42], and microcapsule‐based systems [43]. These materials respond to thermal stimuli through mechanisms such as thermal shutdown, heat absorption, or interfacial self‐healing, thereby delaying thermal propagation [44, 45]. However, such approaches primarily modulate heat generation or transport rather than directly intervening in combustion. Moreover, their response kinetics are often insufficient to cope with the rapid progression of thermal runaway, resulting in delayed or incomplete suppression of fire hazards. These limitations highlight the need for electrolyte systems capable of responding promptly under thermal stimulation [46] and mitigating combustion more effectively.

Herein, we report a dual‐functional fire‐safe microcapsule‐enabled strategy to construct hierarchical solid polymer electrolytes (HSPEs) for LMBs (Figure 1). In this design, perfluoro(2‐methyl‐3‐pentanone) (PFHK) fire‐extinguishing agents are encapsulated within ammonium hydrogen phytate (AHP)‐modified urea‐formaldehyde‐based resin shells and subsequently embedded into an ethylene‐vinyl acetate‐based solid polymer electrolyte (SPE). This hierarchical architecture combines flame‐retardant shielding with thermally triggered fire suppression within the electrolyte framework (Figure 1a). Unlike previously reported microcapsule‐based safety strategies that generally focus on either flame retardancy or fire suppression, the present design integrates both functions within a single thermally responsive microcapsule system. Upon thermal stimulation, the AHP‐modified shell promotes the formation of a protective carbonaceous barrier that delays flame propagation at the initial stage, while further thermal activation triggers PFHK release for active fire suppression (Figure 1b). This sequential and complementary safety response provides hierarchical protection during different stages of thermal abuse. Importantly, this safety‐oriented design does not compromise electrochemical performance. The resulting HSPE exhibits a room‐temperature ionic conductivity of 0.64 mS cm^−1^ and an electrochemical stability window of up to 4.6 V. Symmetric Li||Li cells deliver stable cycling over 325 h, while Li||LFP full cells maintain 81.4% capacity retention upon extended cycling. Furthermore, pouch‐cell tests demonstrate effective suppression of ignition under thermal abuse conditions. This work provides a viable strategy for integrating fire‐suppression functionality with efficient ion transport, offering new insights into the design of safer solid‐state lithium metal batteries.

**FIGURE 1:**
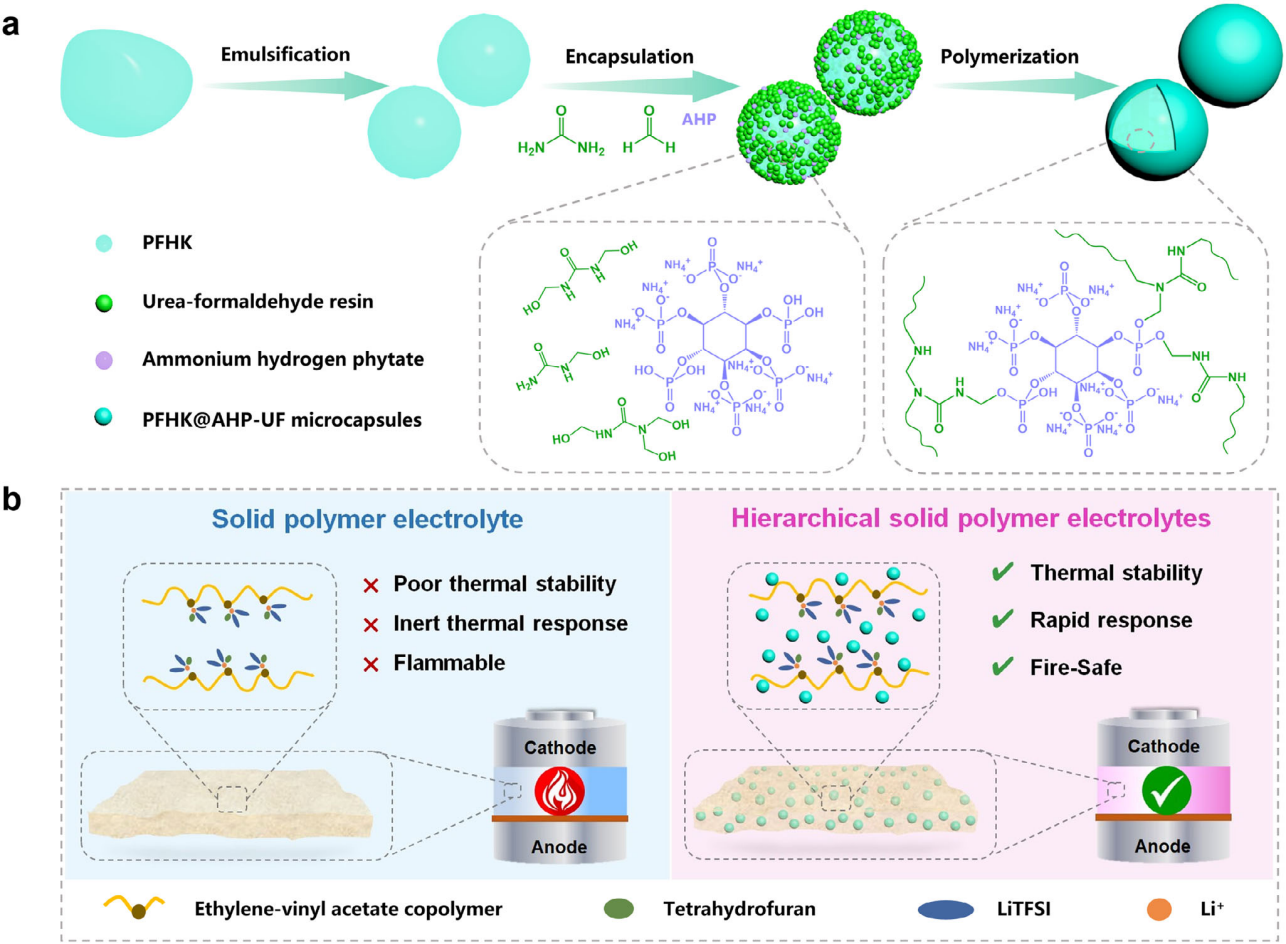
(a) Schematic illustration of fabrication of dual‐functional fire‐safe microcapsules. (b) Schematic illustration of EVA‐based solid polymer electrolytes and hierarchical solid polymer electrolytes, and their integration into lithium metal batteries.

## 2 Results and Discussion

### 2.1 Design and Construction of Dual‐Functional Fire‐Safe Microcapsules

Briefly, the dual‐functional microcapsules (Figure 2a) were fabricated by encapsulating the PFHK fire‐extinguishing agent through the in situ polymerization of urea‐formaldehyde (UF) resin to form the shell layer [47, 48, 49, 50, 51]. The UF shell provides the structural framework for encapsulating and retaining the liquid PFHK core, while the incorporation of AHP into the shell introduces flame‐retardant functionality. Upon thermal stimulation, the encapsulated PFHK can be released to provide active fire suppression, thereby integrating complementary flame‐retardant and fire‐extinguishing functions within a single microcapsule system. As illustrated in Figure S1, AHP was synthesized through the reaction of phytic acid with urea, thereby introducing ammonium species while preserving the phosphate‐rich framework [52, 53]. To investigate the compositional effect of AHP, a series of samples with different urea‐to‐phytic acid molar ratios were prepared and denoted as AHP‐*m*, where *m* represents the corresponding molar ratio. The chemical structures of these AHP samples were examined by FTIR spectroscopy (Figure 2b). Characteristic bands located at ∼940 and 1050 cm^−1^ are assigned to P = O and P‐O stretching vibrations derived from phytic acid, while the band centered at ∼1400 cm^−1^ corresponds to ammonium‐related vibrations [54]. These features confirm successful conversion of phytic acid into ammonium hydrogen phytate. With increasing urea content, the ammonium‐associated absorption progressively intensifies, indicating increased nitrogen incorporation into the phosphorus‐rich molecular structure.

**FIGURE 2:**
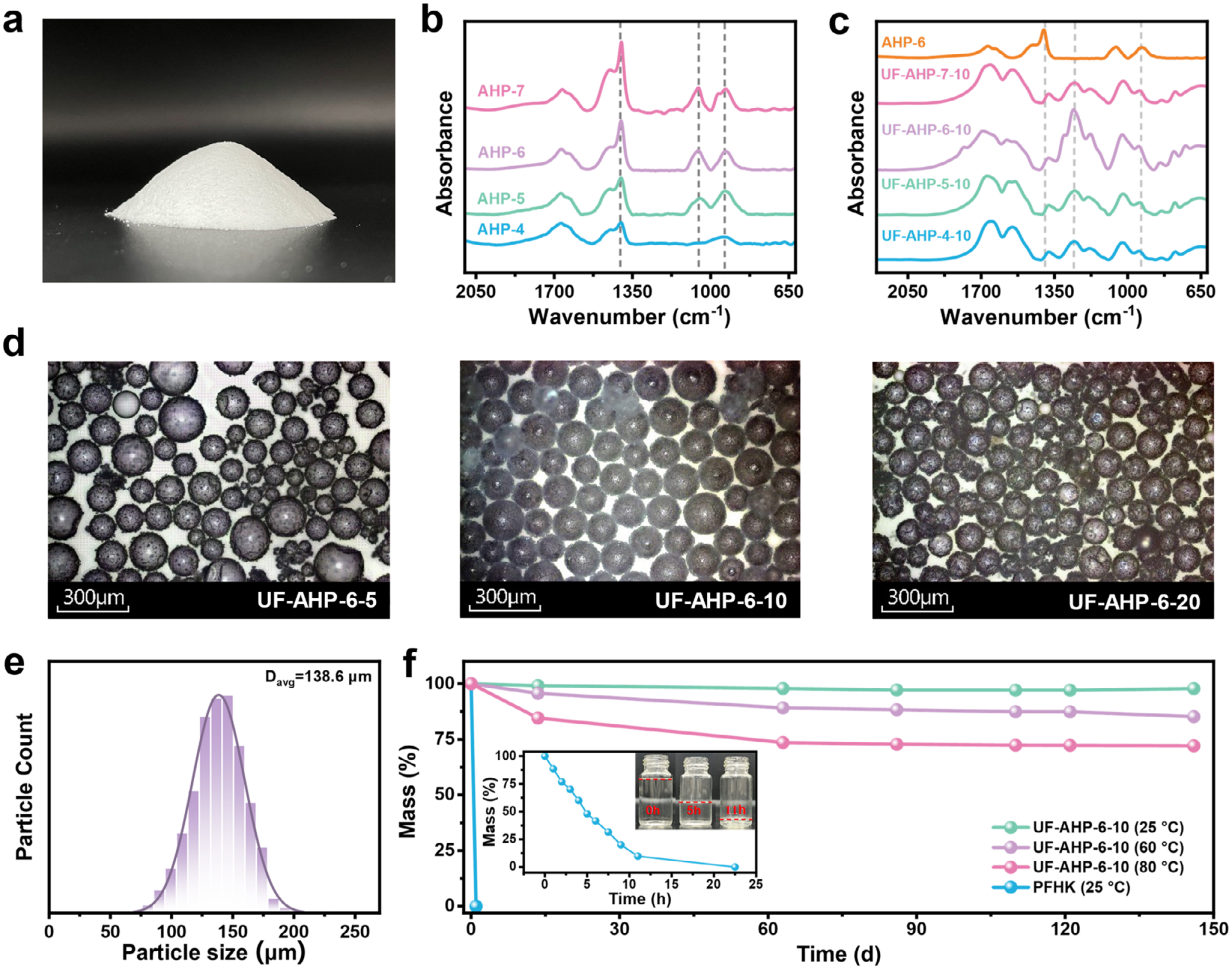
Synthesis and characterization of PFHK@AHP‐UF microcapsules. (a) Macroscopic images of the microcapsules. (b) FTIR spectra of AHP samples with different compositions. (c) FTIR spectra of microcapsules prepared with different AHP compositions. (d) Optical microscopy images of microcapsules prepared with varying AHP‐6 contents. (e) Particle size distribution of UF‐AHP‐6‐10. (f) Mass retention of UF‐AHP‐6‐10 during storage at different temperatures; inset: mass loss of PFHK stored in an open glass vial at room temperature.

The obtained AHP materials were subsequently introduced into the shell‐forming process to fabricate PFHK‐loaded dual‐functional microcapsules, denoted as UF‐AHP‐*m*‐*n*, where *n* represents the mass fraction of urea in the polymerization feed replaced by AHP. For initial screening, *n* was fixed at 10 wt.%. FTIR spectra of the microcapsules (Figure 2c) display characteristic absorptions associated with phosphorus‐containing groups (∼940 cm^−1^), ammonium species (∼1400 cm^−1^), and methylene linkages from the polymer shell (∼1260 cm^−1^), confirming successful incorporation of AHP into the shell matrix. Notably, the intensities of the characteristic bands at ∼940 and ∼1400 cm^−1^ show a clear dependence on the AHP composition. With increasing urea‐to‐phytic acid ratio, the corresponding peak intensities gradually increase, followed by a decrease at higher ratios. A similar variation is observed for the ‐CH_2_‐ vibration band, indicating that the AHP composition influences both the phosphorus/nitrogen functionality and the shell‐forming process. Among the investigated samples, UF‐AHP‐6‐10 exhibits comparatively stronger characteristic signals together with stable shell formation, suggesting that AHP‐6 provides a suitable composition for constructing AHP‐modified UF shells.

Optical microscopy reveals that all microcapsules maintain a well‐defined spherical morphology with good dispersibility (Figures S2 and S3), indicating that AHP incorporation does not disrupt the overall encapsulation process. Nevertheless, the particle size distribution varies markedly with AHP composition. At lower AHP compositions, the microcapsules show relatively broad size distributions, whereas intermediate compositions exhibit improved uniformity. In particular, UF‐AHP‐6‐10 presents a concentrated particle‐size distribution within 100–175 µm, with an average diameter of 138.6 µm. Further increasing the urea‐to‐phytic acid ratio broadens the distribution again, suggesting that the shell‐forming process is sensitive to the chemical composition of AHP. Based on the FTIR and morphological results, AHP‐6 was selected as the preferred AHP composition for subsequent loading optimization.

To further optimize the shell formulation, the loading amount of AHP‐6 was varied while maintaining the same synthetic route. As shown in Figure 2d and Figure S4, all samples retain spherical structures, although their dispersion state and size uniformity evolve with increasing AHP‐6 loading. The pristine UF microcapsules show a broad particle distribution of 75–300 µm, with an average size of 141.2 µm. Introducing 5 wt.% AHP‐6 decreases the average diameter to 109.4 µm, while the size distribution remains relatively broad (Figure S5). Increasing the AHP‐6 content to 10 wt.% results in a larger average diameter of 138.6 µm, accompanied by the most concentrated particle size distribution and optimal morphological integrity. By contrast, further increasing the loading to 20 wt.% reduces the average diameter to 105.9 µm, and irregular, partially fragmented particles begin to appear, indicating excessive disruption of shell growth.

Thermogravimetric analysis further supports the influence of AHP‐6 loading on the thermal characteristics of the microcapsules (Figure S6). Compared with pristine UF microcapsules, all AHP‐6‐modified samples exhibit higher residual mass after thermal decomposition, and the residue gradually increases with increasing AHP‐6 loading. In addition, a mass‐loss stage centered at approximately 125.1°C is observed for all modified samples, which is associated with thermally responsive volatile components from the shell/core system. Although the initial weight‐loss behavior remains generally similar, the high‐temperature decomposition profiles become progressively distinct with increasing AHP‐6 loading [55]. Among the investigated formulations, UF‐AHP‐6‐10 exhibits a relatively high PFHK encapsulation content of 23.9 wt.% together with balanced thermal stability. Based on the combined results of shell morphology, particle‐size distribution (Figure 2e), PFHK encapsulation content (Figure S6c), and thermogravimetric analysis, UF‐AHP‐6‐10 was selected as the representative microcapsule system for subsequent studies.

Given the intrinsic volatility of PFHK, the storage stability of the optimized microcapsules was evaluated by monitoring mass retention at different temperatures (25, 60, and 80°C) to assess the effectiveness of encapsulation in suppressing evaporation. As shown in the inset of Figure 2f, pure PFHK volatilizes rapidly under ambient conditions and undergoes complete mass loss within 23 h, highlighting its limited practical storage stability in the absence of encapsulation. In sharp contrast, UF‐AHP‐6‐10 retains more than 98% of its initial mass after storage for 150 days at 25°C, demonstrating the effective barrier function of the UF‐AHP shell against premature evaporation of the fire‐extinguishing agent. At elevated temperatures, noticeable mass loss occurs during the initial stage, followed by gradual stabilization over prolonged exposure. Specifically, the residual mass stabilizes at approximately 85%–90% at 60°C and ∼75% at 80°C. This temperature‐dependent behavior indicates that PFHK remains well preserved under ambient conditions, while partial release can be triggered under thermal stimulation, which is beneficial for practical battery safety applications requiring both storage stability and rapid fire response.

### 2.2 Fire Response Behavior of Dual‐Functional UF‐AHP Microcapsules

The fire‐response behavior of the optimized microcapsules was first evaluated using self‐supported capsule sheets (50 mm × 50 mm × 2 mm). As shown in Figure 3a, the pristine UF microcapsule sample ignites rapidly upon direct flame contact and undergoes continuous combustion, accompanied by pronounced surface carbonization and progressive erosion of the flame‐exposed region. After prolonged heating, the charred layer is gradually consumed, and renewed ignition occurs when fresh areas are exposed to the flame, implying that the residue generated from the pristine UF shell is discontinuous and insufficient to suppress heat transfer or flame propagation [56]. In contrast, the UF‐AHP‐6‐10 sample exhibits markedly different combustion behavior (Figure 3b). During the initial stage of flame exposure, only slight surface darkening is observed without obvious burn‐through. With continued heating, a continuous carbonaceous layer gradually forms on the exposed surface, effectively resisting further flame penetration. No sustained ignition or re‐ignition is observed throughout the test, and combustion remains localized without spreading across the sample. The preserved structural outline after burning further suggests that the AHP‐modified shell can generate a structurally stable protective layer during combustion [57, 58].

**FIGURE 3:**
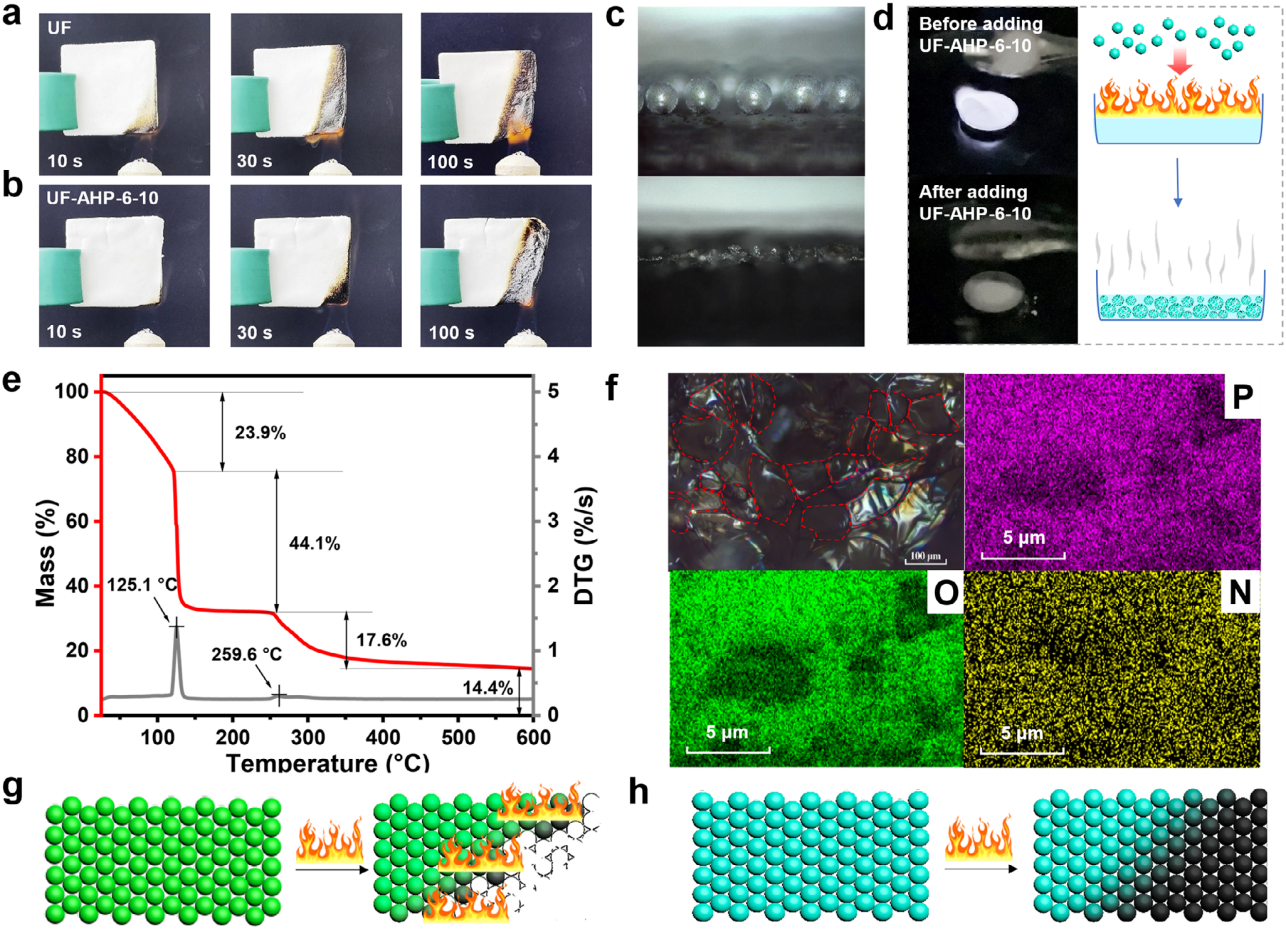
Thermal response, fire‐extinguishing behavior, and underlying mechanism of the microcapsules. Combustion behavior of (a) UF and (b) UF‐AHP‐6‐10 microcapsule sheets under continuous flame exposure. (c) Optical microscopy images of UF‐AHP‐6‐10 before and after thermal rupture. (d) Combustion behavior of a flammable solvent with and without UF‐AHP‐6‐10 microcapsules, together with schematic illustration. (e) Thermogravimetric and derivative thermogravimetric (DTG) curves of UF and UF‐AHP‐6‐10 microcapsules. (f) SEM images of carbonized residues and corresponding EDS elemental mapping (P, O, N). (g,h) Schematic illustration of the fire‐response mechanisms of pristine UF and AHP‐modified microcapsules.

To clarify whether the microcapsules can respond to thermal stimuli, their structural evolution was examined by optical microscopy. As shown in Figure 3c, the initially well‐defined spherical microcapsules collapse into fragmented residues after thermal treatment, indicating rupture of the shell structure. This rapid morphological transition suggests that heating induces shell failure and enables release of the encapsulated PFHK fire‐extinguishing agent. Such behavior is likely associated with internal pressure buildup within the volatile core, together with thermal softening of the shell matrix [59, 60]. The fire‐suppression capability of this thermally triggered release was further examined using a flammable‐solvent ignition test (Figure 3d). Upon flame exposure, the introduced microcapsules are rapidly activated, leading to immediate extinguishment of the burning solvent. The prompt suppression behavior indicates that PFHK release proceeds on a timescale compatible with rapid combustion events, allowing the microcapsules to function as active fire‐response units rather than passive thermal additives.

The thermal decomposition behavior of the microcapsules was further analyzed by thermogravimetric measurements (Figure 3e and Figure S6). Both pristine UF and UF‐AHP‐6‐10 microcapsules exhibit multistep mass‐loss processes associated with moisture evaporation, release of the encapsulated PFHK, and subsequent decomposition of the polymer shell. However, the final residue of UF‐AHP‐6‐10 reaches 15.6 wt.% at 500°C and remains at 14.4 wt.% at 600°C, which is substantially higher than that of pristine UF at 500°C (0.3 wt.%). This enhanced char yield confirms that incorporation of AHP effectively promotes carbonization during thermal degradation.

Post‐combustion morphology provides further insight into the protective mechanism. As shown in the scanning electron microscopy (SEM) images in Figure 3f, the carbonized microcapsules largely retain their shell‐derived framework after release of the core material. The collapsed residues overlap to form a porous and interconnected carbon layer with abundant internal voids. Such a structure effectively retards heat conduction and limits oxygen diffusion during combustion. Elemental mapping further reveals the enrichment of phosphorus‐, nitrogen‐, and oxygen‐containing species within the residue, suggesting the formation of heteroatom‐rich protective char structures.

These above results collectively indicate a dual‐functional fire‐response mechanism (Figure 3g,h). In the pristine UF system, the shell decomposes rapidly and fails to generate an effective barrier, thereby allowing continuous burning. By contrast, the AHP‐modified shell undergoes in situ carbonization to form a compact protective layer that suppresses heat and mass transfer. Meanwhile, rupture of the shell releases PFHK into the surrounding flame zone, providing an additional gas‐phase extinguishing effect. The synergistic coupling of condensed‐phase shielding and active fire suppression accounts for the outstanding fire resistance of the optimized microcapsules.

### 2.3 Construction and Properties of Hierarchical Solid Polymer Electrolytes

The optimized dual‐functional microcapsules were incorporated into an EVA‐based polymer matrix to construct HSPEs [61, 62]. A series of HSPEs were prepared with UF‐AHP‐6‐10 microcapsule loadings of 10, 20, and 30 wt.% relative to the LiTFSI content in the electrolyte, denoted as HSPE‐10, HSPE‐20, and HSPE‐30, respectively, while the microcapsule‐free electrolyte was defined as SPE. The thicknesses of the resulting electrolyte membranes were also characterized, with the corresponding measurements shown in Figure S7. As shown in Figure 4a, the pristine SPE forms a white semi‐transparent membrane with a homogeneous appearance, reflecting the good film‐forming capability of the ethylene‐vinyl acetate copolymer. The membrane exhibits excellent flexibility and mechanical integrity, consistent with the intrinsic elasticity of the polymer matrix. After incorporation of microcapsules, the HSPE membranes become progressively more opaquer and thicker with increasing loading, suggesting the successful introduction of dispersed capsule domains throughout the membrane. Nevertheless, all HSPE membranes remain bendable without visible cracking, suggesting that the continuous polymer framework is largely preserved despite the hierarchical incorporation of solid inclusions. A similar trend is observed in the tensile results shown in Figure 4b. SPE exhibits the highest tensile strength and toughness among all samples. Increasing microcapsule content leads to a gradual decrease in tensile strength, likely arising from partial disruption of matrix continuity and localized stress concentration around the embedded capsules. Notably, HSPE‐10 shows increased elongation at break despite reduced strength, indicating enhanced ductility at moderate loading. At higher capsule contents, both tensile strength and elongation decrease, reflecting increased structural heterogeneity and reduced deformability.

**FIGURE 4:**
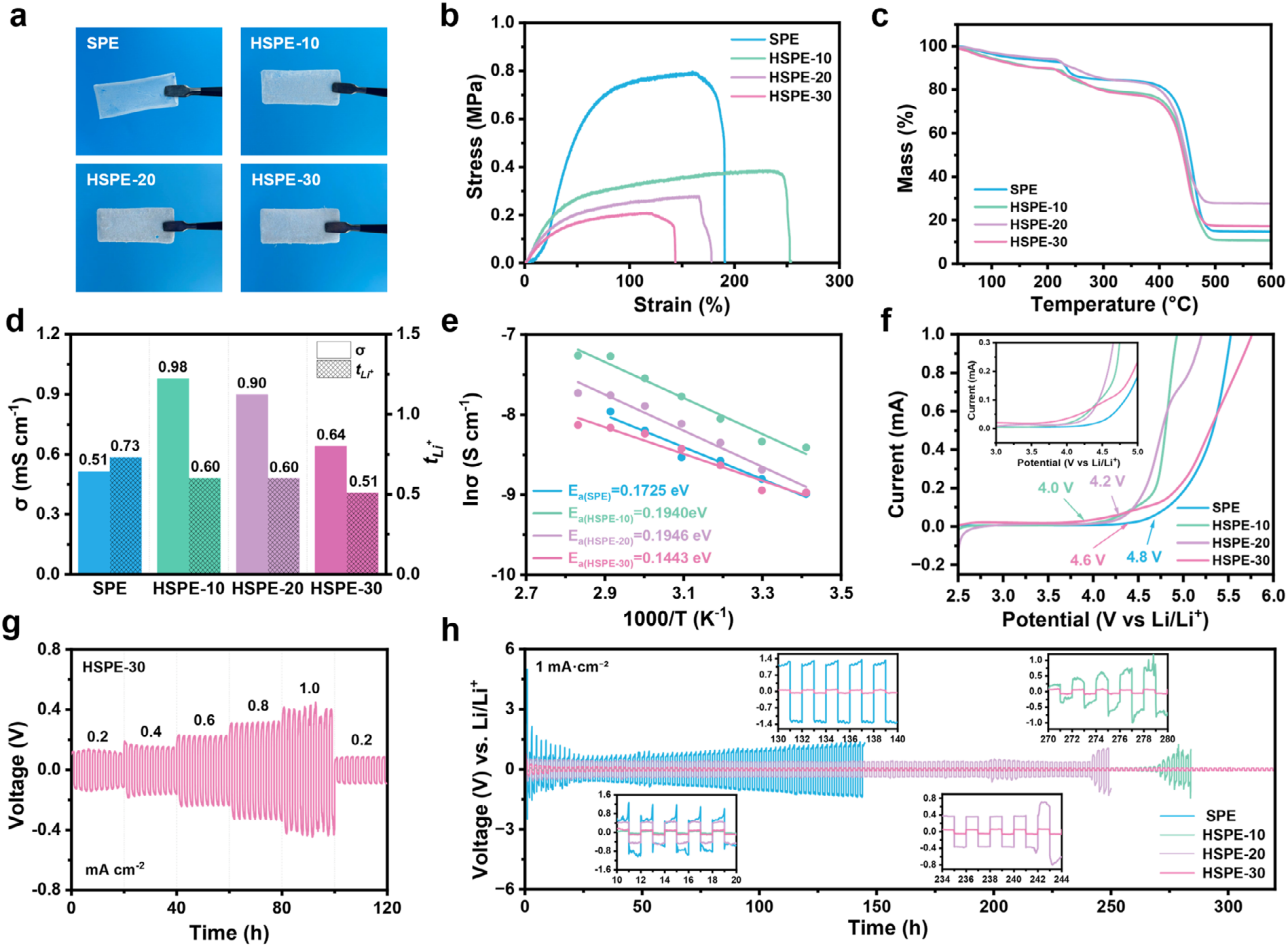
Structural and electrochemical properties of hierarchical solid polymer electrolytes. (a) Digital photographs of pristine SPE and HSPE membranes with varying microcapsule contents. (b) Tensile properties of SPE and HSPE membranes. (c) Thermogravimetric curves of SPE and HSPE membranes. (d) Ionic conductivity and Li^+^ transference number of SPE and HSPEs. (e) Temperature‐dependent ionic conductivity and corresponding Arrhenius plots. (f) LSV curves showing the electrochemical stability window. (g) Cycling performance of Li||Li symmetric cells at various current densities. (h) Li||Li symmetric cell cycling performance at 1 mA cm^−2^.

FTIR spectra further suggest the structural compatibility of the HSPE membranes (Figure S8). All samples display nearly identical characteristic absorption bands, indicating that microcapsule incorporation does not alter the chemical structure of the EVA matrix. The peaks at 2920 and 2850 cm^−1^ correspond to asymmetric and symmetric stretching vibrations of ‐CH_2_ groups, while the band at ∼1740 cm^−1^ arises from C = O stretching of acetate moieties. Additional absorptions in the 600‐1000 cm^−1^ region are associated with C‐O vibrations of EVA and contributions from the LiTFSI anion [63]. With increasing microcapsule content, the peak intensities decrease slightly without obvious band shifts, suggesting physical dispersion of the microcapsules within the polymer matrix rather than chemical interaction. The absence of new characteristic bands further supports good structural compatibility within the hierarchical electrolyte system.

Thermal analysis reveals that incorporation of microcapsules alters the degradation behavior of the membranes (Figure 4c). The pristine SPE exhibits two major decomposition stages centered at approximately 228 and 455°C, leaving a final residue of 14.7 wt.% at 600°C. At low loading, HSPE‐10 shows an additional weight‐loss peak at ∼145°C, associated with volatilization of low‐molecular‐weight species released from the microcapsules, while the final residue decreases to 10.7 wt.%. Increasing the loading to 20 wt.% introduces an additional DTG feature at ∼263°C, which can be associated with decomposition and carbonization of the AHP‐modified shell, accompanied by an increased residue of 17.2 wt.%. Further increasing the capsule content to 30 wt.% yields a higher residue of 27.7 wt.%, indicating the enhanced char‐forming capability introduced by the AHP‐modified microcapsules. It should be noted that the microcapsule loading is defined relative to the LiTFSI content rather than the total electrolyte mass; therefore, the residue of the composite electrolyte cannot be estimated by directly adding the residue fractions of pristine SPE and isolated microcapsules. Moreover, interactions among the electrolyte components during thermal degradation may further alter the carbonization behavior (Figure S9).

The ionic conductivity and lithium‐ion transference number of the electrolytes are summarized in Figure 4d, with the full transference measurements provided in Figure S10. SPE exhibits a room‐temperature ionic conductivity of 0.51 mS cm^−1^. Upon introducing microcapsules, the conductivity increases markedly to 0.98 mS cm^−1^ for HSPE‐10. The relatively high ionic conductivity of HSPE‐10 may be attributed to an appropriate microcapsule content that preserves sufficiently continuous ion‐transport pathways within the polymer electrolyte matrix [43, 64]. In contrast, further increasing the microcapsule content may disrupt the continuity of the ion‐conducting polymer phase and increase the tortuosity of ion‐transport pathways, thereby introducing additional transport resistance and decreasing the ionic conductivity. Further increasing the capsule content gradually decreases the ionic conductivity to 0.90 and 0.64 mS cm^−1^ for HSPE‐20 and HSPE‐30, respectively, although both values remain higher than that of SPE. This non‐monotonic behavior suggests that moderate microcapsule incorporation may facilitate ion transport, whereas excessive loading introduces additional tortuosity and transport resistance within the composite matrix. The lithium‐ion transference number also shows a systematic dependence on capsule loading. The pristine SPE exhibits the highest *t*_Li+_ value of 0.73, indicating a relatively larger fraction of mobile lithium ions in the homogeneous polymer electrolyte. Upon incorporation of microcapsules, the *t*_Li+_ value decreases to 0.60 for both HSPE‐10 and HSPE‐20, and further declines to 0.51 for HSPE‐30. This trend suggests that introduction of the microcapsule phase alters the local ionic transport environment and gradually changes the relative contribution of lithium‐ion migration. Notably, ionic conductivity and the lithium‐ion transference number reflect different aspects of ion‐transport behavior. The decrease in *t_Li+_* with increasing microcapsule content suggests a change in the relative contribution of Li^+^ to the total ionic transport. Because *t_Li+_* represents the fraction of ionic current carried by Li^+^ rather than the magnitude of the total ionic conductivity, a relatively high ionic conductivity can coexist with a lower *t_Li+_*.

The temperature‐dependent ionic conductivity (Figure 4e) exhibits a similar dependence on microcapsule loading. HSPE‐10 maintains the highest conductivity throughout the tested temperature range, followed by HSPE‐20, while SPE and HSPE‐30 exhibit a slight crossover at lower temperatures. At elevated temperatures, SPE displays higher conductivity than HSPE‐30; however, it also undergoes visible swelling and deformation, suggesting limited thermal dimensional stability. In contrast, all HSPE membranes remain structurally intact under identical conditions. Arrhenius fitting yields activation energies of 0.1725 eV for SPE and 0.1443 eV for HSPE‐30, while HSPE‐10 and HSPE‐20 exhibit slightly higher values (>0.19 eV). Despite the lower activation energy of HSPE‐30, its conductivity remains lower than that of HSPE‐10 and HSPE‐20 over most of the temperature range, suggesting that ion transport is governed not only by local migration barriers but also by the spatial distribution of microcapsule domains and the continuity of conductive pathways within the polymer matrix.

The electrochemical stability of the electrolytes was evaluated by linear sweep voltammetry (LSV), with the corresponding curves shown in Figure 4f. The pristine SPE exhibits the highest oxidative stability, with a decomposition onset at 4.8 V. HSPE‐10 shows a lower onset at 4.0 V, while HSPE‐20 and HSPE‐30 exhibit intermediate values of 4.2 and 4.6 V, respectively. Despite this variation, all electrolytes exhibit electrochemical stability windows of at least 4.0 V, suggesting that incorporation of the functional microcapsules maintains a sufficiently wide electrochemical window for practical battery operation [65]. The slightly reduced oxidative stability of HSPE‐10 compared with SPE may be associated with changes in the local interfacial chemical environment introduced by microcapsule incorporation. To further investigate the surface chemical environments of the cycled electrolytes, high‐resolution XPS measurements were performed on SPE and HSPE‐10 (Figures S11–S15). No distinct P 2p signal was resolved under the present detection conditions, providing no direct evidence for significant surface accumulation of P‐containing species. The C 1s, F 1s, N 1s, and O 1s spectra show the characteristic chemical environments of the electrolyte components, with differences in their relative contributions after microcapsule incorporation. In the F 1s spectra, both samples exhibit F‐C‐ and F‐Li‐related components; however, these signals cannot be uniquely assigned to PFHK because fluorine‐containing electrolyte species also contribute to the F 1s response. These results provide additional information on the surface chemical environments after cycling and complement the electrochemical and interfacial analyses [66, 67].

The interfacial compatibility with lithium metal was further examined using Li||Li symmetric cells. During stepwise current cycling from 0.2 to 1.0 mA cm^−2^ and back (Figure 4g and Figure S16), HSPE‐30 exhibits lower and more stable overpotentials than SPE, particularly at elevated current densities where SPE shows a rapid increase in polarization. This reduced polarization under dynamic cycling conditions suggests a more stable lithium deposition/stripping process in the hierarchical electrolyte system. Under prolonged cycling at 0.1 mA cm^−2^ (Figure S17a), the overpotential of SPE gradually increases, indicating increased interfacial polarization during repeated plating/stripping. In contrast, all HSPE cells exhibit substantially more stable polarization, with HSPE‐30 exhibiting the most stable long‐term response. Similar behavior is observed at 0.2 mA cm^−2^ (Figure S17b). When the current density is increased to 1.0 mA cm^−2^ (Figure 4h), SPE suffers from severe polarization, whereas HSPE‐30 retains significantly lower overpotentials and exhibits stable cycling over 325 h. These results collectively demonstrate that incorporation of the dual‐functional microcapsules improves interfacial stability with lithium metal while maintaining robust ion transport under practical operating conditions. Post‐cycling SEM observations further reveal a relatively uniform morphology of the HSPE‐30‐based Li electrode, with less pronounced large‐scale agglomeration and no obvious dendritic or needle‐like Li growth (Figures S18 and S19). Together with the stable polarization behavior during prolonged Li plating/stripping, these observations further support the improved interfacial stability of HSPE‐30 with lithium metal.

### 2.4 Fire Safety Performance of HSPEs

To evaluate the fire safety of the electrolytes, open‐flame combustion tests were carried out on SPE and HSPE membranes with different microcapsule loadings (Figure 5a–d). Upon direct exposure to an external flame, the pristine SPE ignites immediately and undergoes vigorous sustained combustion even after removal of the ignition source, accompanied by a bright orange‐yellow flame. The burning process persists for approximately 55 s, during which continuous material consumption is observed, reflecting the intrinsically flammable nature of the polymer matrix. After incorporation of the dual‐functional microcapsules, the combustion behavior changes significantly in a loading‐dependent manner. At a relatively low loading (HSPE‐10), ignition still occurs, but the flame intensity is noticeably weakened and partial self‐extinguishing behavior is observed during combustion. Increasing the microcapsule content to 20 wt.% (i.e., HSPE‐20) leads to intermittent flame extinction during exposure, and the membrane is able to self‐extinguish shortly after ignition, suggesting effective suppression of sustained burning. In sharp contrast, HSPE‐30 cannot be ignited under identical conditions, even after 15 repeated exposures to the flame. Instead of continuous combustion, a rapidly formed carbonized surface layer is observed, which acts as a barrier to heat transfer and flame propagation across the membrane [68]. These results demonstrate that the incorporation of dual‐functional microcapsules endows HSPEs with integrated flame‐retardant and active fire‐extinguishing functions. Under thermal abuse conditions, the AHP‐modified shell promotes the formation of a protective carbonaceous barrier, while thermally triggered PFHK release provides active fire suppression. The complementary action of these two functions effectively suppresses combustion and enhances the safety of HSPEs under thermal abuse conditions.

**FIGURE 5:**
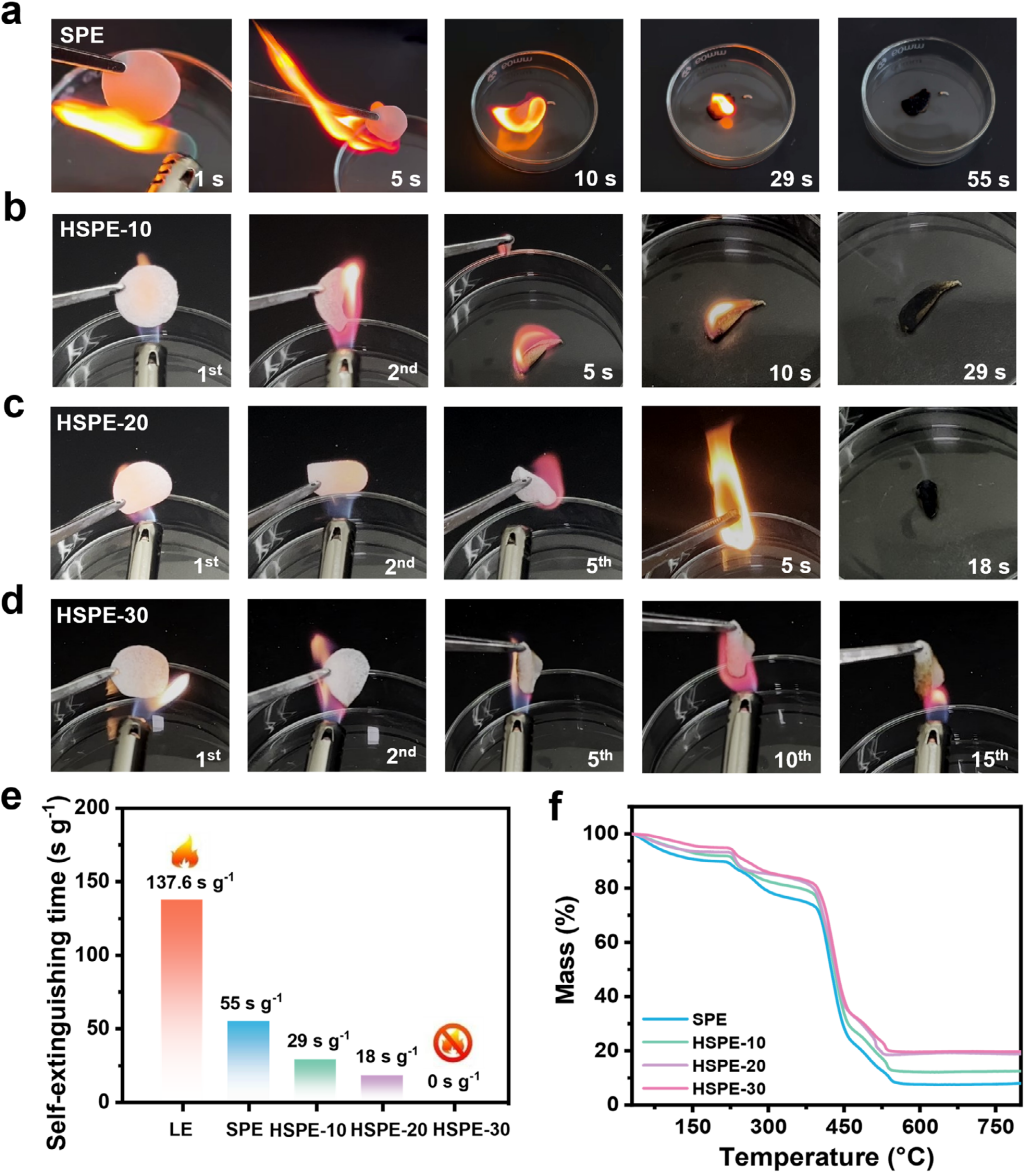
Fire safety and flame‐retardant properties of hierarchical electrolytes. (a) Combustion behavior of pristine SPE under open‐flame exposure. (b–d) Combustion behavior of HSPEs with varying UF‐AHP‐6‐10 microcapsule loadings. (e) Self‐extinguishing time of SPE and HSPEs. (f) Residual char of SPE and HSPEs after combustion.

The evolution in combustion behavior can be further understood by comparison with a control electrolyte containing unmodified UF microcapsules (Figure S20). Although release of PFHK after shell rupture provides transient flame suppression, sustained burning is still observed because the pristine UF shell lacks intrinsic flame‐retardant functionality. By contrast, HSPEs containing AHP‐modified microcapsules exhibit more pronounced surface carbonization during flame exposure. This behavior is associated with thermal decomposition of AHP, which generates phosphorus‐ and nitrogen‐containing species that promote condensed‐phase char formation [69]. With increasing microcapsule loading, the progressively strengthened protective layer ultimately leads to complete ignition suppression in HSPE‐30. Quantitative evaluation of self‐extinguishing behavior further supports this trend (Figure 5e). The pristine liquid electrolyte exhibits a self‐extinguishing time [70, 71, 72] of 137.6 s g^−1^, while SPE shows a reduced value of 55 s g^−1^. Intermediate HSPE compositions display further decreases to 29 and 18 s g^−1^ for HSPE‐10 and HSPE‐20, respectively. Notably, HSPE‐30 exhibits a self‐extinguishing time of 0 s g^−1^, indicating complete non‐flammability under the tested conditions. These results indicate a gradual transition from highly flammable to fire‐resistant behavior with increasing microcapsule incorporation.

To quantify the condensed‐phase protection effect, the residual char content after combustion was measured (Figure 5f). A monotonic increase in carbon residue is observed with increasing microcapsule loading. HSPE‐30 exhibits the highest char yield of 19.7 wt.%, which is substantially higher than that of pristine SPE (7.9 wt.%). This enhanced residue formation suggests that incorporation of AHP promotes the formation of a compact phosphorus/nitrogen‐rich protective layer during combustion. Such a layer serves as an effective thermal and physical barrier, suppressing heat feedback and limiting oxygen diffusion, thereby inhibiting further flame propagation.

Post‐combustion thermal decomposition analysis (Figure S21) provides additional insight into the fire‐protection mechanism. The pristine SPE shows a minor weight‐loss event near 225°C and a dominant decomposition stage between 370 and 450°C. After incorporation of microcapsules, a new decomposition feature appears at approximately 260°C, corresponding to degradation and carbonization of the microcapsule shell. The intensity of this peak increases with capsule loading, while the main decomposition rate in the 370–450°C region gradually decreases. Meanwhile, the high‐temperature decomposition tail above 500°C becomes progressively suppressed. These changes suggest that early formation of a protective char layer alters the degradation pathway of the polymer matrix, delays high‐temperature pyrolysis, and improves structural stability during combustion.

### 2.5 Electrochemical Performance of HSPE‐Based LMBs

Considering the electrochemical and safety performance of HSPE membranes with different microcapsule contents, HSPE‐30 was selected for subsequent full‐cell evaluation because it provides a favorable overall balance between ionic transport capability, electrochemical stability, and safety performance. The HSPE membranes were used as solid‐state electrolytes in Li||LFP full cells to evaluate there electrochemical performance. The cells were cycled within a voltage window of 2.5–4.0 V. Figure 6a presents the charge–discharge profiles of the HSPE‐30‐based Li||LFP cell during the first 100 cycles at 0.1 C, while the corresponding curves of the SPE‐based cell under identical conditions are provided in Figure S22. Both systems deliver comparable reversible capacities, and the voltage plateau positions together with their evolution during cycling remain similar. No obvious collapse of the discharge plateau or severe polarization growth is observed in either system during the initial cycles. Compared with SPE, the HSPE‐30‐based cell shows slightly less smooth voltage profiles, manifested as small but more frequent local fluctuations along the charge/discharge plateaus. Post‐cycling EIS measurements reveal increased interfacial impedance (Figure S23), suggesting that gradual interfacial evolution during prolonged cycling may contribute to the observed local voltage fluctuations and increased polarization.

**FIGURE 6:**
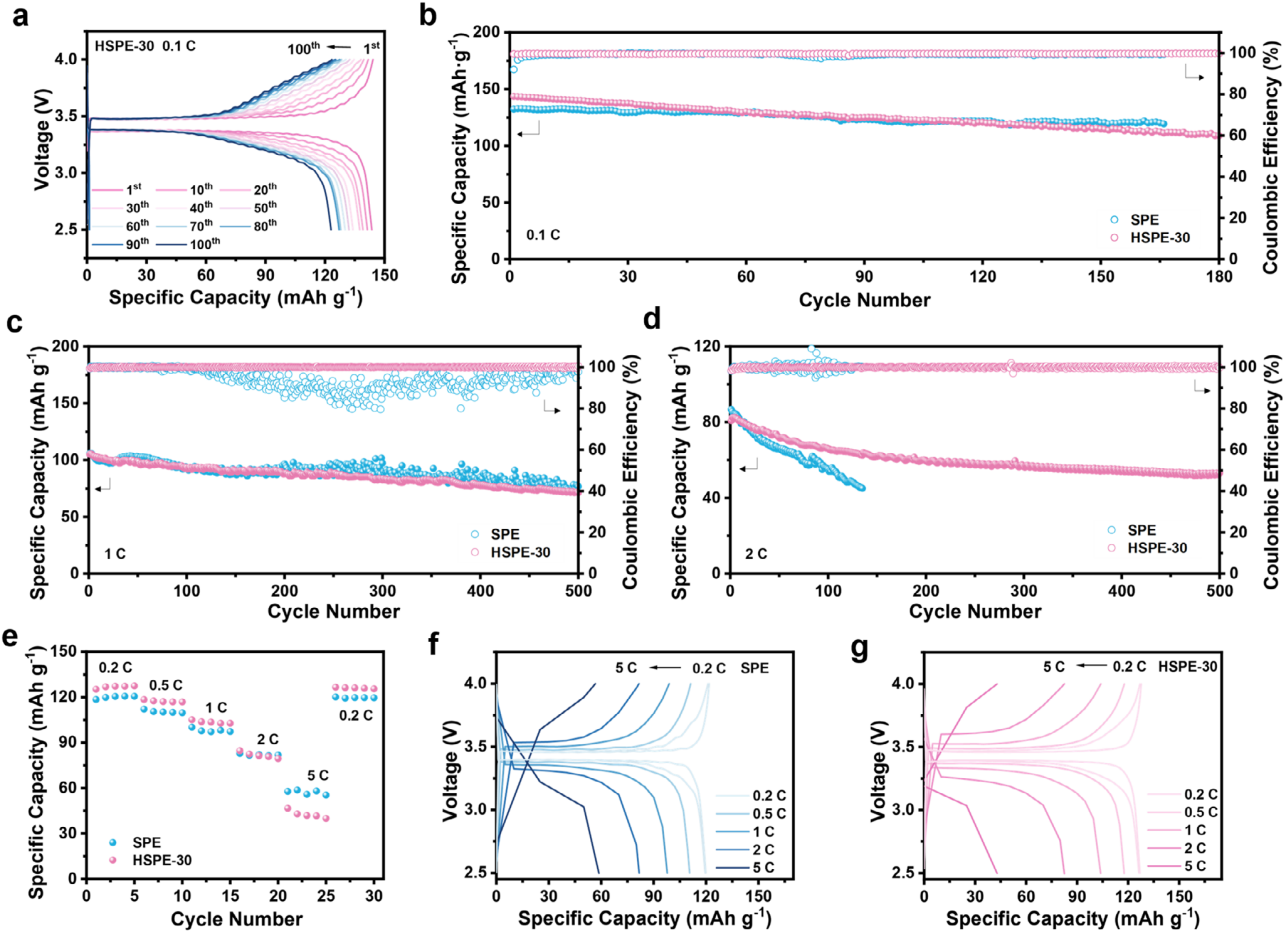
Electrochemical performance of Li||LFP cells based on HSPE electrolytes. (a) Charge–discharge profiles of the HSPE‐30‐based cell during the first 100 cycles at 0.1 C. (b–d) Long‐term cycling performance of Li||LFP cells with SPE and HSPE‐30 at 0.1 C, 1 C, and 2 C, respectively. (e) Rate performance of Li||LFP cells with SPE and HSPE‐30. (f,g) Charge‐discharge profiles of Li||LFP cells with SPE and HSPE‐30 at different current densities.

The long‐term cycling stability derived from these charge–discharge profiles is summarized in Figure 6b. The HSPE‐30‐based cell maintains stable discharge capacity during prolonged cycling at 0.1 C without abrupt capacity decay, retaining approximately 114 mAh g^−1^ after 180 cycles, corresponding to a capacity retention of 81.4%. The voltage polarization between charge and discharge plateaus remains relatively small, although a slight gradual increase is observed upon extended cycling, suggesting slowly evolving interfacial resistance during long‐term operation. To further assess durability under higher current densities, long‐term cycling tests were conducted at 1 C and 2 C (Figure 6c,d). At 1 C, the HSPE‐30‐based cell delivers an initial discharge capacity of 105 mAh g^−1^, which gradually decreases to 78 mAh g^−1^ after 500 cycles (74.3% retention). The coulombic efficiency remains consistently above 99.9% throughout cycling without noticeable fluctuation. At 2 C, the initial capacity is 80 mAh g^−1^, with 54 mAh g^−1^ retained after 500 cycles (67.5% retention). The cycling profiles remain smooth and stable, without abrupt voltage or capacity fluctuations. In contrast, although the SPE‐based cell exhibits a slightly higher initial capacity at 2 C, it undergoes rapid capacity fading, retaining only approximately 65% of its initial capacity within 100 cycles. This difference indicates the superior structural and interfacial stability of the hierarchical electrolyte under demanding operating conditions.

The rate capability of the HSPE‐30‐based cell is presented in Figure 6e, where the current rate is sequentially increased from 0.2 to 5 C. A gradual decrease in discharge capacity is observed with increasing rate, consistent with enhanced polarization and increasingly limited ion‐transport kinetics under high‐current operation. When the current rate is returned to 0.2 C, the capacity largely recovers to its initial level, suggesting that the electrolyte‐electrode interfaces remain stable during repeated high‐rate cycling. At relatively low current densities (0.2–1 C), the HSPE‐30‐based cell delivers slightly higher discharge capacities than the SPE‐based counterpart. This difference becomes less pronounced as the current rate increases, where transport limitations dominate the cell response. The corresponding charge‐discharge profiles (Figure 6f,g) show that HSPE‐30 maintains well‐defined voltage plateaus across different rates, while only mild plateau broadening appears at high current densities, reflecting acceptable kinetic polarization during fast lithium‐ion transport.

To further demonstrate practical applicability, pouch cells were assembled and subjected to mechanical damage followed by direct flame exposure (Figure 7a,b). The SPE pouch cell ignites immediately when flame is applied to the damaged region, followed by sustained combustion and rapid flame propagation. In contrast, the HSPE‐30 pouch cell does not exhibit sustained ignition under identical conditions, and only localized surface carbonization is observed during flame exposure, demonstrating effective suppression of thermal runaway propagation at the device level. The electrochemical performance of the HSPE‐30 pouch cell is shown in Figure 7c. At 0.2 C, the cell delivers an initial coulombic efficiency of 99.1% and retains 97.7% of its capacity after 200 cycles. The corresponding charge‐discharge profiles remain stable throughout cycling without obvious deformation of the voltage plateaus, indicating robust electrochemical reversibility in the pouch‐cell configuration. As shown in Figure 7d, the charged pouch cell is capable of powering multiple LEDs simultaneously, and the light emission remains stable under bending deformation. Even after mechanical cutting, the brightness decreases but the external circuit remains functional, indicating that partial electrochemical output can still be maintained under structural damage conditions. These results show that the HSPE‐based pouch cell can maintain electrochemical functionality under mechanical deformation, reflecting the mechanical robustness of the electrolyte system. Its improved fire‐resistant behavior further supports the safety advantages of HSPEs under mechanical and thermal abuse conditions.

**FIGURE 7:**
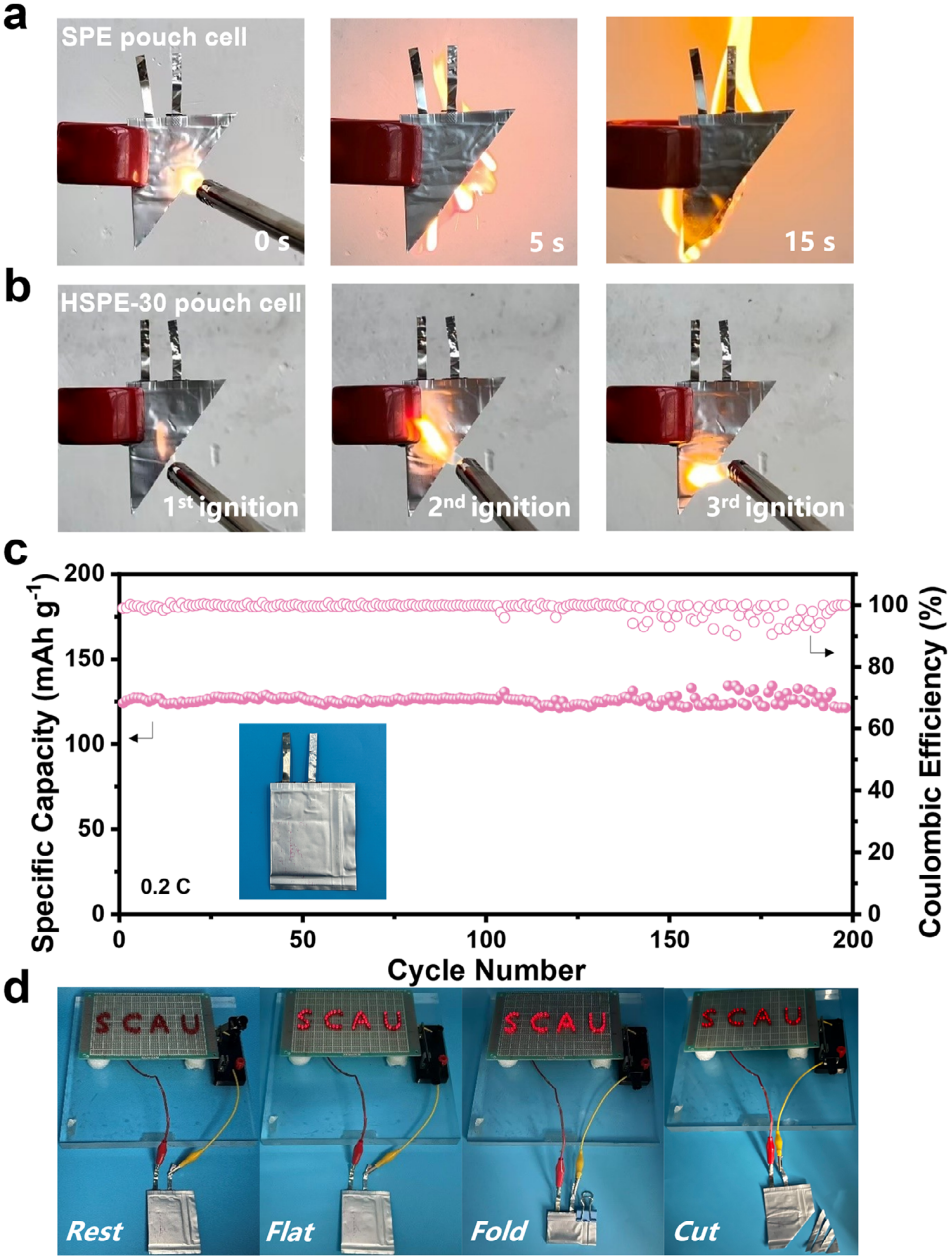
Safety performance and electrochemical behavior of pouch cells based on HSPE electrolytes. (a,b) Flame response of SPE and HSPE‐30 pouch cells under direct flame exposure. (c) Cycling performance of the HSPE‐30 pouch cell at 0.2 C. (d) Photographs of the HSPE‐30 pouch cell powering LEDs under normal, bent, and cut conditions.

## 3 Conclusions

In summary, we developed a hierarchical solid polymer electrolyte incorporating dual‐functional fire‐safe microcapsules for lithium metal batteries. By encapsulating PFHK within AHP‐modified urea‐formaldehyde shells and incorporating these units into an EVA‐based electrolyte, the resulting HSPE combines electrochemical functionality with enhanced fire safety. The microcapsules enable long‐term retention of the fire‐extinguishing agent under ambient conditions, while rapid release can be triggered by thermal stimulation. Meanwhile, the phosphorus/nitrogen‐containing shell promotes the formation of a protective char layer, thereby coupling condensed‐phase protection with active fire suppression. The resulting HSPE‐30 membrane delivers a room‐temperature ionic conductivity of 0.64 mS cm^−1^, stable lithium plating/stripping behavior, and durable Li||LFP full‐cell performance, retaining 74.3% and 67.5% of the initial capacity after 500 cycles at 1 C and 2 C, respectively. The resulting electrolyte remains nonflammable under open‐flame exposure, while pouch cells resist sustained ignition even after mechanical damage. The overall fabrication strategy relies on readily available raw materials and conventional microencapsulation and solution‐casting processes, suggesting its potential for further process optimization and scale‐up. This work highlights a safety‐by‐design strategy for integrating complementary flame‐retardant and active fire‐suppression functions within polymer electrolytes, providing multistage protection against thermal abuse and offering a promising pathway toward safer high‐energy batteries.

## Author Contributions

**Ziqi Huang**: methodology, software, formal analysis, data curation, visualization, writing – original draft, writing – review and editing, investigation, validation. **Jiuqing Gui**: methodology, validation, investigation, writing – original draft, visualization, formal analysis, software, data curation. **Qiaoying Cao**: project administration, formal analysis, data curation, supervision, writing – review and editing. **Yong Xiao**: formal analysis, data curation, project administration, methodology. **Hang Hu**: formal analysis, project administration, data curation, methodology. **Yeru Liang**: writing – review and editing, project administration, resources, supervision, funding acquisition, investigation, conceptualization.

## Conflicts of Interest

The authors declare no conflicts of interest.

## Supporting information




**Supporting File**: advs78034‐sup‐0001‐SuppMat.docx.

## Data Availability

The data that supports the findings of this study are available in the supplementary material of this article.
