# Supplementary material for: Fire‐Safe Hierarchical Solid Polymer Electrolytes Enabled by Dual‐Functional Microcapsules for Lithium Metal Batteries
Source: Adv Sci (Weinh). 2026 Oct 4:e78034. Online ahead of print. doi: 10.1002/advs.78034 (PMC13634469; doi:10.1002/advs.78034)
Supplement: Supplementary file 1 [file ADVS-9999-e78034-s001.docx]

Supporting Information

**Fire-Safe Hierarchical Solid Polymer Electrolytes Enabled by Dual-Functional Microcapsules for Lithium Metal Batteries**

*Ziqi Huang, Jiuqing Gui, Qiaoying Cao, Yong Xiao, Hang Hu, Yeru Liang^*^*

Key Laboratory for Biobased Materials and Energy of Ministry of Education, College of Materials and Chemical Engineering, South China Agricultural University, Guangzhou 510642, P. R. China

Email: liangyr@scau.edu.cn

**Experimental Section**

**Materials**

All chemicals were used as received without further purification unless otherwise specified. Urea, formaldehyde solution, sodium hydroxide (NaOH), phytic acid (70 wt%), lithium bis-(trifluoromethanesulfonyl)imide (LiTFSI, 99%), N-methyl-2-pyrrolidone (NMP), and tetrahydrofuran (THF) were purchased from Aladdin Biochemical Technology Co., Ltd. Perfluorooctanesulfonic acid (99%) was obtained from Morinaga Chemical Co., Ltd. Perfluorohexanone (99.7%) was supplied by Zhejiang Noah Fluorochemical Co., Ltd. Lithium iron phosphate (LFP, battery grade) was purchased from Shenzhen Kejing Zhida Technology Co., Ltd. Ethylene-vinyl acetate copolymer (EVA, 32 wt% VA) was obtained from Sinopec Maoming Petrochemical Co., Ltd. Acetylene black (Super P, battery grade) and poly(vinylidene fluoride) (PVDF 5130, battery grade) were purchased from Guangdong Zhuguang New Energy Technology Co., Ltd. Ultrapure water was used in all experiments.

**Synthesis of flame-retardant microcapsules**

Ammonium hydrogen phytate (AHP) was synthesized by reacting phytic acid with urea (molar ratio 1:6) at 120 °C for 4 h, followed by purification and redissolution to obtain a 70 wt% aqueous solution. The urea-formaldehyde (UF) resin prepolymer was prepared by reacting urea with formaldehyde (37 wt%) at pH 8.5-9.5 and 70 °C for 1 h. A perfluorohexanone emulsion was obtained by dispersing perfluorohexanone in an aqueous solution of a perfluorinated surfactant under stirring. The UF prepolymer was mixed with the emulsion under mechanical stirring, and AHP solution was added to adjust the pH to 3.0-3.5, initiating in situ polymerization. The reaction was maintained at 25 °C for 6 h and subsequently heated to 60 °C for 2 h. The resulting microcapsules were collected, washed, and dried.

**Synthesis of hierarchical solid polymer electrolytes**

LiTFSI was dissolved in THF, followed by the addition of EVA at a mass ratio of 1:1. The mixture was stirred for 24 h and sonicated at 40 °C for 1 h to obtain a homogeneous solution, which was then cast onto a glass substrate and dried at room temperature to constant weight, yielding a pristine solid polymer electrolyte (SPE). Hierarchical solid polymer electrolytes (HSPEs) were prepared by introducing optimized UF-AHP-6-10 flame-retardant microcapsules into the homogeneous solution under stirring, followed by casting and drying under identical conditions to obtain composite electrolyte films. The thicknesses of the obtained electrolyte membranes were measured using a digital micrometer. The incorporation of microcapsules leads to an increase in membrane thickness due to the introduction of the dispersed microcapsule phase within the polymer matrix. The SPE, HSPE-10, HSPE-20, and HSPE-30 membranes exhibited thicknesses of approximately 0.19, 0.34, 0.40, and 0.46 mm, respectively (Figure S7).

**Preparation of LFP cathodes and cell assembly**

The LFP cathode was prepared by a conventional slurry-casting method. LFP, Super P, and PVDF were mixed at a mass ratio of 8:1:1. PVDF was dissolved in NMP and added to the LFP/Super P mixture, followed by stirring to obtain a homogeneous slurry. The slurry was cast onto aluminum foil and dried at 80 °C under vacuum for 12 h. The electrodes were punched into 12 mm disks for coin cell assembly, and the active material loading was recorded for electrochemical testing.

All solid-state lithium metal coin cells were assembled in an argon-filled glovebox with O_2_ and H_2_O levels below 0.1 ppm (CR2032-type configuration). The cells were stacked in the order of stainless-steel case, spring, spacer, cathode, SPE or HSPE membrane, lithium metal anode, and top case. The lithium metal anode and electrolyte membrane were punched into 16 mm and 19 mm disks, respectively. All assembled coin cells were pressed and rested at room temperature for 12 h prior to testing. No liquid electrolyte was used.

For pouch cell assembly, the LFP cathode was cut into 20 × 20 mm electrodes, while lithium metal anodes (25 × 25 mm) and solid-state electrolyte membranes (30 × 30 mm) were prepared accordingly. Pouch cells were assembled in an argon-filled glovebox (O_2_ and H_2_O < 0.1 ppm) using aluminum-plastic laminated films. The cell was constructed in a stacked configuration of current collector, lithium metal anode, solid-state electrolyte, and LFP cathode to ensure intimate interfacial contact. No liquid electrolyte was introduced.

**Characterizations**

The morphology and size distribution of the microcapsules were observed using an optical microscope (Axio Imager A2m, Carl Zeiss). Particle size distributions were analyzed using Nano Measurer software based on images collected at ×100 and ×200 magnifications. Storage stability was evaluated by monitoring mass changes of samples stored at 25, 60, and 80 °C at fixed time intervals. Fourier transform infrared spectra were recorded using a Bruker Vertex 70 spectrometer in the range of 4000-500 cm^-1^, with samples prepared as KBr pellets at a mass ratio of 1: 100. Thermogravimetric analysis was conducted under nitrogen atmosphere from 25 to 600 °C to evaluate the thermal stability, core content, and char residue of the microcapsules and solid-state electrolytes. Additional thermogravimetric analysis measurements were performed under air atmosphere from 25 to 800 °C to assess the combustion residue (carbon content) of the electrolytes. The morphology and elemental composition of combustion residues were characterized using field-emission scanning electron microscopy (SU8220, Hitachi) coupled with energy-dispersive X-ray spectroscopy (Oxford Instruments).

Flame extinguishing performance was evaluated by igniting 200 μL of flammable organic electrolyte in a metal dish with and without flame-retardant microcapsules, and flame behavior was recorded using a digital camera. Flame retardancy was further assessed by exposing samples (50 × 50 × 2 mm) to an alcohol lamp flame and recording burning behavior.

Mechanical properties were measured using a universal testing machine (UTM4204, Sansi Materials Testing Co., Ltd.) at a tensile rate of 10 mm min^-1^ using rectangular samples with dimensions of 50 × 20 mm. Electrochemical performance was evaluated using an electrochemical workstation (CHI660E), and charge-discharge tests were conducted using a battery testing system (Neware BTS 7.6.x).

**Electrochemical characterization of electrolytes**

The ionic conductivity of the solid polymer electrolyte was evaluated using electrochemical impedance spectroscopy (EIS) on a CHI660E workstation. Symmetric stainless-steel blocking cells were assembled by sandwiching the electrolyte between two stainless-steel electrodes. EIS was performed in the frequency range of 10^6^ to 10^-2^ Hz with an AC amplitude of 5 mV, and temperature-dependent measurements were conducted using the same configuration. The ionic conductivity ($\sigma$) was calculated from the bulk resistance according to:

$\sigma=\frac{d}{A\cdot R}$	（1）

where *d* is the electrolyte thickness, *A* is the electrode area, and *R* is the bulk resistance obtained from Nyquist plots. The lithium-ion transference number (*t_Li⁺_*) was determined using DC polarization combined with EIS in symmetric Li||electrolyte||Li cells. The transference number was calculated using:

$t_{{Li}^{+}}=\frac{I_{S}(\Delta V-I_{0}R_{0})}{I_{0}(\Delta V-I_{S}R_{S})}$	（2）

where *I_0_* and *I_s_* are the initial and steady-state currents, respectively, *ΔV* is the applied DC polarization voltage (10 mV), and *R_0_* and *R_s_* represent the interfacial resistances before and after polarization, respectively, obtained from electrochemical impedance spectroscopy (EIS). The electrochemical stability window was evaluated by linear sweep voltammetry (LSV) from 2 to 6 V (vs. Li^+^/Li) at a scan rate of 0.1 mV s^-1^, with decomposition onset identified by a sharp current increase.

**Electrochemical performance of batteries**

Galvanostatic charge-discharge measurements were conducted using a Neware BTS 7.6.x system. Symmetric cells were tested at current densities of 0.1-1 mA cm^-2^, while LFP full cells were evaluated within a voltage window of 2.5-4.0 V (vs. Li^+^/Li). EIS of full cells was conducted under identical conditions as described above. LFP served as the cathode and lithium metal as the anode. Nyquist plots were used to analyze bulk resistance, charge transfer resistance, and ion diffusion behavior.

**Figure S1.** Synthesis of ammonium hydrogen phytate.

**Figure S2.** Optical microscopy images of microcapsules prepared with varying urea-to-phytic acid molar ratios.

**Figure S3.** Particle size distribution of the microcapsules prepared with varying urea-to-phytic acid molar ratios.

**Figure S4.** Optical microscopy image of UF microcapsules prepared without AHP-6.

**Figure S5.** Particle size distributions of microcapsules prepared with varying AHP-6 contents.

**Figure S6.** Thermogravimetric analysis of microcapsules prepared with varying AHP-6 contents.

**Figure S7.** Thickness measurement of SPE and HSPE membranes.

**Figure S8.** FTIR spectra of SPE, HSPE-10, HSPE-20, and HSPE-30.

**Figure S9.** Thermogravimetric and derivative thermogravimetric (DTG) curves of SPE, HSPE-10, HSPE-20, and HSPE-30 electrolytes.

**Figure S10.** Lithium-ion transference number of SPE, HSPE-10, HSPE-20, and HSPE-30.

**Figure S11.** High-resolution XPS spectra of C 1s for SPE and HSPE-10 electrolytes after cycling.

**Figure S12.** High-resolution XPS spectra of F 1s for SPE and HSPE-10 electrolytes after cycling.

**Figure S13.** High-resolution XPS spectra of O 1s for SPE and HSPE-10 electrolytes after cycling.

**Figure S14.** High-resolution XPS spectra of N 1s for SPE and HSPE-10 electrolytes after cycling.

**Figure S15.** High-resolution XPS spectra of P 2p for SPE and HSPE-10 electrolytes after cycling.

**Figure S16.** Cycling performance of Li||Li symmetric cells with SPE electrolyte at various current densities.

**Figure S17.** Cycling performance of Li||Li symmetric cells with SPE, HSPE-10, HSPE-20, and HSPE-30 electrolytes at current densities of (a) 0.1 mA cm^-2^ and (b) 0.2 mA cm^-2^.

**Figure S18.** Post-cycling SEM images of the Li metal anode from the SPE-based Li||Li symmetric cell: (a) cross-sectional morphology and (b) surface morphology.

**Figure S19.** Post-cycling SEM images of the Li metal anode from the HSPE-30-based Li||Li symmetric cell: (a) cross-sectional morphology and (b) surface morphology.

**Figure S20.** Combustion behavior of HSPEs containing UF microcapsules without AHP modification: (a) HSPE with 10 wt% UF microcapsules, (b) HSPE with 20 wt% UF microcapsules, and (c) HSPE with 30 wt% UF microcapsules.

**Figure S21.** DTG curves of different electrolytes after combustion testing.

**Figure S22.** Charge-discharge profiles of the SPE-based Li||LFP cell during the first 100 cycles at 0.1 C.

**Figure S23.** Electrochemical impedance spectra of (a) SPE and (b) HSPE-30 measured before and after cycling.
